# Responses of Purple Rice Genotypes to Nitrogen and Zinc Fertilizer Application on Grain Yield, Nitrogen, Zinc, and Anthocyanin Concentration

**DOI:** 10.3390/plants10081717

**Published:** 2021-08-20

**Authors:** Suksan Fongfon, Chanakan Prom-u-thai, Tonapha Pusadee, Sansanee Jamjod

**Affiliations:** 1 Department of Plant and Soil Sciences, Faculty of Agriculture, Chiang Mai University, Chiang Mai 50200, Thailand; suksan_fo@cmu.ac.th (S.F.); tonapha.p@cmu.ac.th (T.P.); 2 Lanna Rice Research Center, Chiang Mai University, Chiang Mai 50200, Thailand; 3 Innovative Agriculture Research Center, Faculty of Agriculture, Chiang Mai University, Chiang Mai 50200, Thailand

**Keywords:** purple rice, zinc fertilizer, nitrogen fertilizer, biofortification, anthocyanin

## Abstract

Purple rice is recognized as a staple food for humans and as a source of anthocyanins and micronutrients such as zinc (Zn). This study examined how nitrogen (N) and Zn fertilizers affected grain yield and grain N, Zn, and anthocyanin concentration among purple rice genotypes. Six purple rice genotypes (PIZ, KAK, KS, KH-CMU, KDK, and HN) were grown under two levels of N, the optimum N60 (60 kg/ha) and high N180 (180 kg/ha) rates, along with three Zn application methods (no Zn application (Zn0), soil Zn application (ZnS; 50 kg ZnSO_4_/ha), and foliar Zn spray (ZnF; 0.5% ZnSO_4_ at the rate of 900 L/ha three times at heading, flowering, and early milk stages). Grain yield of the five purple rice landraces increased by 21–40% when increasing N from N60 to N180, although no response was found with HN. The higher N rate increased grain N concentration by 10–50% among the genotypes, while anthocyanin concentration increased by 100–110% in KAK and KS, and grain Zn was increased in KS. Applying ZnS increased grain yield by 16–94% but decreased anthocyanin and N concentrations compared to the control Zn0. Applying ZnF effectively increased grain Zn concentration by 40–140% in the genotypes without adversely impacting grain anthocyanin or N concentration. This study demonstrated that the appropriate management of N and Zn fertilizers for specific purple rice genotypes would be one way to increase productivity and grain N, Zn, and anthocyanin concentration.

## 1. Introduction

Purple rice is recognized as a functional food for promoting human health, as it is a potential source of various bioactive compounds and micronutrients [1,2]. There are a variety of bioactive substances found in purple rice, including phenolic acid, flavonoids, anthocyanin, proanthocyanin, tocopherols, tocotrienols, and gamma oryzanol [3]. Anthocyanin is the major bioactive compound in purple rice, being present in high concentration compared to red or colorless rice [4]. Purple rice has attracted attention from health-conscious consumers because of its antioxidant and anti-inflammatory properties [5,6]. The antioxidants in purple rice help to prevent serious diseases such as cancers, including breast, liver, oral, and colon cancers, with effects involving inhibition of cancer cell invasion and tumor cell growth [7,8,9,10]. Consumption of purple rice also contributes to lower risks of coronary heart disease and diabetes [11,12]. Purple rice is not restricted to being consumed as a staple food, its medicinal and pharmaceutical properties have led to use as an ingredient in dietary supplements and cosmetic products [13].

Additionally, purple rice not only provides antioxidants but also contains carbohydrates, proteins, and micronutrients such as Zn [14]. Zinc is one of the essential micronutrient nutrients necessary for both physical and mental development [15]. An insufficient Zn intake can negatively impact human health, especially among infants where it causes higher risk for infectious diseases, diarrhea, and growth retardation [16,17]. In children and adolescents, Zn deficiency has been reported to be involved in deficits in neuropsychologic functioning and activity or motor development and thus interferes with cognitive performance [18]. Alternatively, symptoms in the elderly were related to taste disorders, loss of appetite, lowered immune response, and delayed wound healing [19,20]. Moreover, Zn is of interest in terms of its supporting antioxidants by mediating anti-inflammatory and antioxidant pathways that contribute to the prevention of chronic diseases such as tumorigenesis, cardiovascular disorders, and chronic liver disease [21,22,23,24].

The bioactive compounds and nutrient content of rice grains is one of the major indicators used to evaluate purple rice grain quality. Recent studies have found that grain anthocyanin and Zn concentrations vary by rice genotype. Variation of anthocyanin content in unpolished rice among 13 colored rice genotypes from China, Sri Lanka, and Thailand, ranging from 19.4 to 140.8 mg/100 g was reported [25]. On study found a 25-fold variation in anthocyanin concentration in the bran among 25 purple rice genotypes from the USDA National Small Grains Collection [26]. In another study, 42 pigmented rice genotypes obtained from IRRI reported Zn content ranging from 133–224 mg/kg [27]. In Thailand, 21 selected purple rice landraces in northern Thailand displayed anthocyanin and Zn concentrations of 77.3 ± 22.7 mg/100 g and 20.5 ± 3.0 mg/kg, respectively [28]. In addition to the variation among genotypes, environmental factors have also been reported to strongly affect grain quality. For example, growing purple rice genotypes at different elevations affected grain anthocyanins, Fe, Zn, and their antioxidant capacity by DPPH and FRAP methods [29]. Other factors such as water management during cultivation indirectly affect the grain anthocyanin. It has been reported that growing purple rice in waterlogged conditions improved grain anthocyanin and Fe and Zn concentrations compared to dry-land cultivation, particularly among the genotypes with originally high grain nutritional concentration [30]. However, the effects of N and Zn fertilizer application on grain N, Zn, and anthocyanin concentration of purple rice are less well characterized compared to colorless rice.

It has been well documented that N fertilization contributes to improving the quality of non-pigmented rice, while it has been reported that applying twice the recommended rate of N promotes synthesis and accumulation bioactive compounds in rice grains such as tocopherols, tocotrienols, and γ-oryzanol [31]. Similar results have been obtained for other grain nutrients such as Fe, Mg, and Zn [32,33]. Zinc biofortification through applying fertilizer was suggested as an effective method to improve grain Zn in rice, especially via foliar and soil application [34,35]. However, limited information is available concerning the efficacy of Zn fertilizer application in combination with varied N fertilizer conditions on grain anthocyanin, the major bioactive compound, grain N, and Zn among purple rice genotypes. We hypothesized that rice genotypes would respond differently to N and Zn fertilizer management due to the variation in yield potential and grain Zn accumulation among the genotypes in response to fertilizer application. Therefore, this study examined the effects of N and Zn fertilizer application on grain yield and grain N, Zn, and anthocyanin concentration in six selected purple rice genotypes. The information will be useful for improving productivity and grain nutritional quality among purple rice genotypes by N and Zn fertilizer management. The results can thus be incorporated into plant breeding programs.

## 2. Results

The results showed significant difference among purple rice genotypes in all characters and interaction effects between the two factors in some characters, but no interaction effects between the three factors (genotype, N rate and Zn application method) was observed in all characters measured in this study (Table 1).

### 2.1. Grain Yield and Yield Components

**Figure 1:**
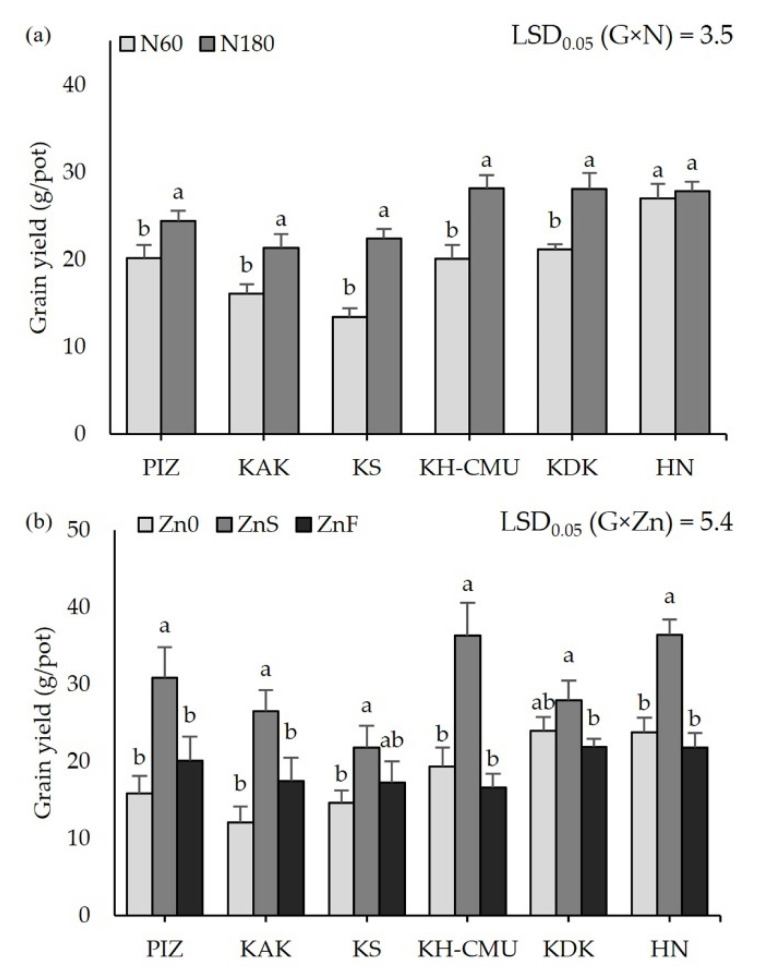
Grain yield of six purple rice genotypes grown under two levels of N application at 60 kg/ha (N60) and 180 kg/ha (N180) (**a**) and three Zn application treatments, no Zn (Zn0), soil Zn (ZnS; 50 kg ZnSO4/ha), and foliar Zn application (ZnF; 0.5% ZnSO4 at the rate of 900 L/ha three times at heading, flowering, and early milk stages) (**b**). The bars represent standard errors of the means (n = 4). Different letters above bars indicate significant differences by the least significant difference (LSD) at *p* < 0.05.

Grain yield among six purple rice genotypes was significantly affected by N fertilizer (*p* < 0.05, Figure 1a). Grain yield varied from 12 g/pot in KS to 27 g/pot in HN when plants were grown under N60, the accepted optimum N application rate. Increasing the N rate from N60 to N180 enhanced grain yield in five genotypes, with increases ranging from 3 to 9 g/pot. The highest increase of grain yield was 75% in KS, and the lowest was 14% in PIZ from N60 application, but no effect of N rate on grain yield was found in HN. In contrast, grain yield was affected by Zn application differently among genotypes regardless of N fertilizer (*p* < 0.05, Figure 1b). Grain yield varied from 15 g/pot in PIZ, KAK, and KS to 24 g/pot in KAK and HN when no Zn (Zn0) was applied. Applying ZnS improved grain yield in almost all genotypes compared to plants grown under Zn0, while grain yield among the genotypes was not affected by ZnF. Grain yield was increased by 40 to 100%, being the highest in PIZ and KH-CMU and lowest in KS under application of ZnS, while grain yield was not affected in KDK.

**Figure 2:**
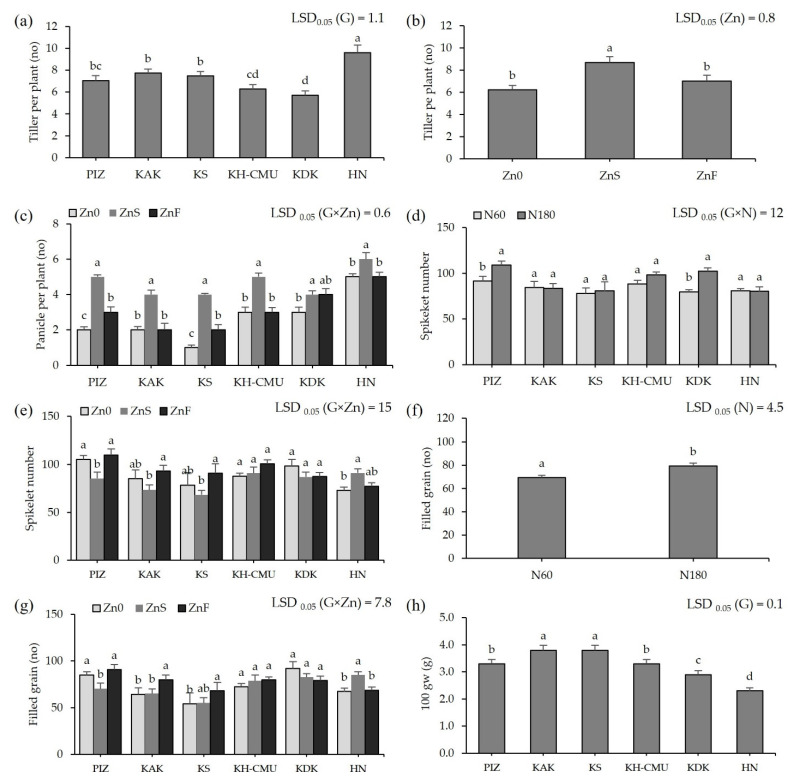
Tiller numbers of six purple rice genotypes (**a**). Tiller numbers under three Zn applications (**b**). Panicle numbers of six purple rice genotypes grown under three Zn applications (**c**). Spikelet numbers of six purple rice genotypes grown under two levels of N (**d**) and three Zn applications (**e**). Filled grain numbers of six purple rice genotypes grown under two levels of N and three Zn applications (**f**,**g**). The hundred grain weights of six purple rice genotypes (**h**). The bars represent standard errors of the means (n = 4). Different letters above bars indicate significant differences by the least significant difference (LSD) at *p* < 0.05.

The average tiller number among the genotypes varied from 6 tillers in KH-CMU and KDK to 10 tillers in HN regardless of the level of N and Zn fertilization (Figure 2a). Applying ZnS increased the number of tillers by approximately 3 tillers, while applying ZnF had no effect on the number of tillers compared to the control Zn0 (Figure 2b). The panicle number among genotypes was affected by Zn application (Figure 2c), but not by N rate. Without applying Zn, the panicle number varied between 1 and 5. Applying ZnS increased the average panicle number of all rice genotypes by 1 to 3 panicles/plant, the highest increase being by 3 in KS and the lowest by 0.2 in HN compared with no Zn application. Applying ZnF increased average panicle number in PIZ and KS by 0.5 and 1, respectively, whereas there was no effect on the other genotypes. The N rates affected the spikelet number among genotypes; the spikelet number varied from 78 spikelets in KS to 91 spikelets in PIZ when plants were grown under N60 (Figure 2d). Higher N rate increased the spikelet number by 28% in KDK and by 19% in PIZ, but no significant effects were found in the other genotypes. In addition, applying ZnS promoted spikelet number in HN by 25% but decreased the number in PIZ by 19%, while the effect was not found in the other genotypes. Applying ZnF did not affect the spikelet number in any of the genotypes (Figure 2e). Filled grain number was affected by Zn application (Figure 2g), but not by N rates. In Zn0, filled grain number varied from 54% to 85% among the six genotypes. Applying ZnS increased filled grains in HN by 24%, but decreased the number in PIZ by 21%, while applying ZnF increased filled grain number in KAK and KS by 26% and 10%, respectively, while the other genotypes were not affected. Increasing N rate improved filled grain among genotypes by 14% (Figure 2f). The hundred grain weight was affected by genotype without an effect from N or Zn application; the highest grain weight was found in KS at 3.8 g, while the lowest grain weight at 2.3 g was found in HN (Figure 2h).

### 2.2. Grain Grain N, Zn, and Anthocyanin Concentration

**Figure 3:**
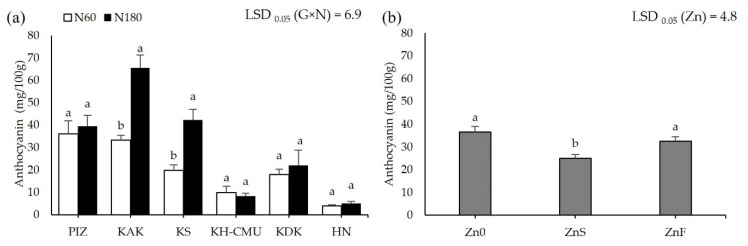
Anthocyanin concentration of six purple rice genotypes grown under two levels of N application, 60 kg/ha (N60) and 180 kg/ha (N180) (**a**), and three Zn application methods (no Zn (Zn0), soil Zn (ZnS; 50 kg ZnSO_4_/ha), and foliar Zn application (ZnF; 0.5% ZnSO_4_ at the rate of 900 L/ha for three times at heading, flowering, and early milk stages) (**b**). The bars represent standard errors of the mean (n = 4). Different letters above bars indicate significant differences by the least significant difference (LSD) at *p* < 0.05.

**Figure 4:**
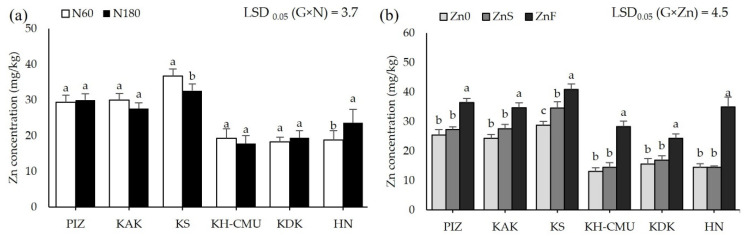
Grain Zn concentrations of six purple rice genotypes grown under two levels of N, 60 kg/ha (N60) and 180 kg/ha (N180) (**a**) and three Zn application methods by no Zn (Zn0), soil Zn (ZnS; 50 kg ZnSO_4_/ha) and foliar Zn application (ZnF; 0.5% ZnSO_4_ at the rate of 900 L/ha for three times at heading, flowering, and early milk stages) (**b**). The bars represent standard errors of the means (n = 4). Different letters above bars indicate significant differences by the least significant difference (LSD) at *p* < 0.05.

**Figure 5:**
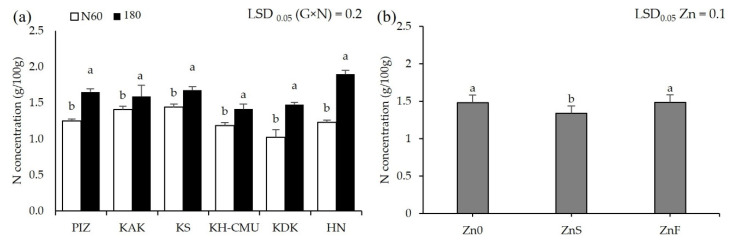
Grain N concentrations of six purple rice genotypes grown under two levels of N (60 kg/ha (N60) and 180 kg/ha (N180)) (**a**) and three Zn application methods, no Zn (Zn0), soil Zn (ZnS; 50 kg ZnSO_4_/ha) and foliar Zn application (ZnF; 0.5% ZnSO_4_ at the rate of 900 L/ha for three times at heading, flowering, and early milk stages) (**b**). The bars represent standard errors of the means (n = 4). Different letters above bars indicate significant differences by the least significant difference (LSD) at *p* < 0.05.

Grain anthocyanin concentration was affected by N rates differently among the genotypes (*p* < 0.05, Figure 3a), but not by Zn application. Grain anthocyanin varied from 4 mg/100 g in HN to 36 mg/100 g in PIZ when plants were grown under N60. Increasing N rate from N60 to N180 increased grain anthocyanin by 100% in KAK and by 110% in KS, while the other genotypes were not affected. Applying ZnS decreased grain anthocyanin by an average 30% among the six genotypes, while applying ZnF did not affect grain anthocyanin compared to the control Zn0 (Figure 3b). Grain Zn was also affected by N and Zn fertilizers differently among genotypes (*p* < 0.05, Figure 4a,b). Plants grown under N60 had grain Zn concentrations from 18.3 mg/kg in KDK to 36.7 mg/kg in KS. Increasing N rate increased grain Zn concentration by 40% in HN, but decreased grain Zn concentration by 12% in KS, while the other genotypes were not affected. Applying ZnS increased grain Zn by 19.8% in KS, while applying ZnF increased grain Zn in all genotypes, from 41% to 142%, with the highest increase in HN and the lowest in KAK. Grain N concentration was affected by N and Zn fertilizer differently among genotypes (*p* < 0.05, Figure 5a,b). Grain N varied from 1.0% to 1.4% among the genotypes grown under N60, while increasing the N rate to N180 enhanced grain N concentration by 13–54% compared to N60. Applying ZnS decreased 13.3% grain N, while applying ZnF did not affect grain N among the genotypes.

### 2.3. Relationship between Grain Yield and Grain N, Zn, and Anthocyanin Concentration

**Figure 6:**
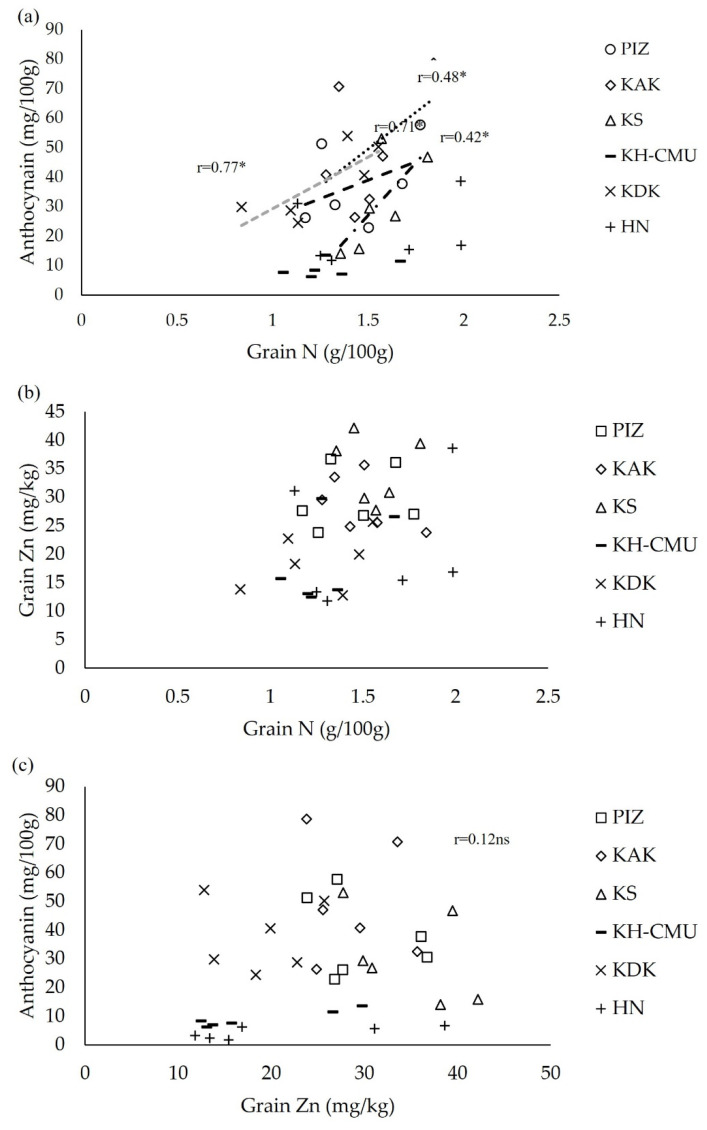
Correlations between grain N and anthocyanin (**a**), between grain N and Zn (**b**), and between grain Zn and anthocyanin (**c**). * indicates significant correlation, and ns represents non-significant difference at *p* < 0.05 (n = 6).

Grain yield was moderately negative correlated with grain anthocyanin in PIZ and HN, but not in the other genotypes. Additionally, grain yield was also weakly to moderately negative correlated with grain Zn concentration in KH-CMU and HN, and grain N in KAK and KH-CMU (Table 2). Concerning the grain N, Zn, and anthocyanin concentration, grain N was positively correlated with anthocyanin in the genotypes KDK, KAK, KS and PIZ, while in HN and KH-CMU there was no relationship (Figure 6a). However, there were no significant correlations between grain N and Zn concentration (Figure 6b) or between grain Zn and anthocyanin concentration in any of the genotypes (Figure 6c).

## 3. Discussion

This study has shown that grain yield and grain N, Zn, and anthocyanin concentration responded differently to N and Zn application among the purple rice genotypes. The five purple rice landraces potentially increased their productivity by 14 to 75% under an increase in the N application rate from 60 to 180 kg/ha, and productivity increased by 40 to100% with soil Zn application, mainly by improving the numbers of tillers and panicles per plant in all genotypes. For grain N, Zn, and anthocyanin concentration, anthocyanin concentration was improved when the higher N rate was applied to KAK and KS, but this was not observed in the other genotypes. Additionally, grain Zn concentration was increased by 41 to 142% among the genotypes by foliar Zn application, but there was no such effect from soil Zn application. Thus, grain yield and grain N, Zn, and anthocyanin concentration can be effectively improved by the management of N and Zn fertilizer in specific purple rice genotypes. However, the results should be further confirmed by conducting similar experiments with greater numbers of rice genotypes displaying variation in yield potential and grain N, Zn, and anthocyanin concentration.

The improvement of rice crops by applying N fertilizer is due to the increase of the yield components in the vegetative and reproductive stages, as found in the present study where the numbers of tillers and panicles per plant were enhanced under higher N application. Many studies have reported that a high rate of N application could promote total chlorophyll content in rice plants as well as the photosynthetic rate and development of spikelets, characteristics that are correlated with grain yield improvement, but the appropriate rate of N varied according to genotype and soil fertility conditions [36,37,38,39]. HN is a purple rice genotype with photoperiod insensitivity, but its yield and yield components were not responsive to the higher N application as observed among the other five landraces. The genotypes may specifically respond to N application rate in their yield production [40]. Therefore, it is necessary to understand how different purple rice genotypes respond to N application rates, especially regarding the photoperiod sensitivity and insensitivity characteristics. The present study has confirmed that soil Zn application effectively enhanced grain yield among the genotypes compared with the foliar Zn application. This was in accordance with a previous study reporting that soil Zn application at 50 kg/ha increased grain yield from 2.1 to 10.2% compared with foliar Zn application with 0.5% ZnSO_4_ that increased grain yield from 0.2 to 4.0%, respectively [41]. Zinc plays a major role in the development of tiller buds in rice plants; the meristem tissue requires 10 times more Zn than mature leaf blades for the functions of cell division and elongation, and thus Zn deficiency can inhibit tiller formation [42,43]. Zn fertilizer enhances photosynthesis through improving the photosynthetic rate and by increasing the chlorophyll content as well as the translocation of photosynthates to the grain [44,45,46]. Thus, applying soil Zn at the early growth stage is more efficient for boosting yield formation than the foliar Zn application in the later stage.

Additionally, applying N and Zn fertilizer not only influences plant growth, development, and productivity but also affects the grain N, Zn, and anthocyanin concentration of rice. This study found that in some purple rice genotypes, e.g., KAK and KS, grain anthocyanin increased in association with grain yield by N application, indicating that applying N could promote anthocyanin biosynthesis and accumulation in rice grains without adversely affecting productivity. A previous study showed that applying N at 120 kg/ha increased anthocyanin in the tubers of purple potatoes [47]. This phenomenon was also observed in red cabbage by applying N at 120–160 kg/ha, depending on plant density [48]. In rice, it was reported that applying N at 120 kg/ha improved anthocyanin content in the leaves and stems of the purple rice genotypes, but not in grains [49]. Thus, grain anthocyanin accumulation response to N application differed among the purple genotypes, as found in this study. The current study has established that increasing grain anthocyanin by N application was correlated with grain N concentration, suggesting that improving grain N concentration by N application may be a key factor in promoting protein synthesis in the pathway of anthocyanin synthesis in plant tissues. This suggestion was supported by a previous study demonstrating that applying N to rice plants increased N accumulation and upregulated the expression of genes involved in anthocyanin synthesis, particularly from the onset of black rice grain development [50]. However, grain anthocyanin in some genotypes did not respond to N application; it would be interesting to investigate this in more detail regarding the physiological mechanisms such as the relationship of N and anthocyanin synthesis pathways and genetic expression by molecular analysis.

Grain Zn concentration in all rice genotypes was improved by foliar Zn application, with the extent of increase depending on the genotype. The result was consistent with a previous study reporting that foliar Zn application increased grain Zn concentration by 25% among different soil fertility profiles of five countries from China, India, Laos PDR, Thailand, and Turkey [34,51]. Additionally, foliar Zn application increased grain Zn by 62.7 and 48.3% in the high and sufficient Zn soils, while soil Zn application increased grain Zn concentration by 15.6 and 5.3%, respectively [41]. A previous study reported that foliar micronutrient application was effective in reducing deficiencies in crop plants due to the nutrients being directly mobilized into the plant tissue without the impacts of soil chemical properties, as is sometime observed in soil fertilizer application [52]. It was also interesting to observe from this study that grain Zn concentration in KS genotypes could be improved by both soil and foliar Zn applications. Thus, the same genotype may have variation in the efficiency of Zn use through absorption, transport, and remobilization, factors that should be further evaluated in various methods of Zn application.

## 4. Materials and Methods

### 4.1. Plant Culture

The pot experiment was conducted in a greenhouse at the Department of Agronomy, Faculty of Agriculture, Chiang Mai University during the wet season (June-November) in 2019. The experiment was arranged in a factorial design in RCBD with 3 factors and 4 replications. Six purple rice genotypes, including Pi Ei Zu (PEZ), Kum Doi Saket (KDK), Kum Hom CMU (KH-CMU), Khao Saeng (KS) Kum Ar Ka (KAK), and Hom Nil (HN) were selected to test in this study. The first 5 genotypes were famous traditional types with high nutritional values and photoperiod sensitivity, while the last genotype was an improved high yield with photoperiod insensitivity. Plants were grown in 14 cm diameter pots containing 3.2 kg soil. The chemical profile of the soil used in the experiments was the Sansai series, a soil with a sandy loam texture. Soil pH 6.5 (1:1, soil:water), organic matter 1.38% (Walkley–Black method); total N 0.07% (Kjeldahl method); available phosphorus 35.06 mg kg^–1^ (Bray II), exchangeable potassium 39.87 mg kg^–1^ (NH_4_OAc, pH 7), and extractable Zn 0.73 mg kg^–1^ (DTPA) [53]. Seven-day-old seedlings were planted, 1 plant per hill, with 3 plants per pot. Two N rates of 60 kg/ha (N60) and 180 kg/ha (N180) were applied equally at the tillering and flowering stages. Three Zn application methods were applied consisting of a control treatment with no Zn applied (Zn0), ZnSO_4_ at 50 kg/ha applied to the soil at tillering stage (ZnS), and Zn foliar spray with 0.5% of ZnSO_4_ at the rate of 900 L/ha 3 times at booting, flowering, and early milk stages (ZnF), approximately ZnSO_4_ was foliar applied at 13.5 kg/ha in total. The growth stage of purple rice referred as in a previous publication [54,55]. All experimental units had the same rates of P and K applied at 60 kg/ha. Plants were grown under waterlogged conditions until maturity. The fungicide Isoprothiolane and insecticide Fipronil were applied at recommended rates to control pests. Weeds were manually removed.

### 4.2. Data Collection

The numbers of tillers were recorded at maximum tillering stage. At the physiological maturity stage, the number of panicles per plant, spikelet number, filled grains per panicle, and hundred grain weight were recorded. Grain yield per pot was determined at a 14% moisture content. Paddy rice was de-husked to yield brown rice for chemical analysis.

### 4.3. Chemical Analysis

All chemicals used in chemical analysis for the concentration of anthocyanin, N, and Zn were analytical grade.

#### 4.3.1. Anthocyanin Determination

Anthocyanin concentration was determined by the pH differential method [56]. Approximately 2.5 g of unpolished rice was extracted for anthocyanin with 24 mL of 70% acidified methanol pH 1.0 and shaking for 60 min, then filtered with Whatman No. 5 filter paper and adjusted to a total volume of 25 mL with 70% acidified methanol pH 1.0 Each 2 mL of extracted solution was prepared with KCl 0.024 M pH 1.0 and CH_3_COONa 0.400 M pH 4.5. The absorbance was measured by spectrophotometer (Biochrom, Libra S22, Cambridge, England) at 520 and 700 nm wavelength.

#### 4.3.2. Zn Determination

The Zn concentration was determined by the dry-ashing protocol. The 0.5 g samples were weighed into a crucible and dry-ashed at 535–540 °C for 8 h. The ash samples were incubated in 1:1 HCl:H_2_O at 70 °C for 20 min, the total volume adjusted to 10 mL with deionized water, and then filtered with Whatman no. 1 filter paper before the Zn concentration was determined with atomic absorption spectrophotometry (Z-8230 Polarize, Zeeman, Hitachi, Japan) [57].

#### 4.3.3. Nitrogen Determination

Nitrogen concentration was analyzed by the Kjeldahl method [58]. A sample of 0.2 g was digested with 5 mL of sulfuric acid (H_2_SO_4_), and the speed and efficiency of the digestion were increased by adding 1.1 g of a catalyst mixture (potassium sulfate: copper sulfate: salicylic acid: selenium 100:10:10:1). The sample was heated during the following step from 120 °C for 20 min to 360 °C for 270 min. The samples were cooled down to room temperature and then diluted with water and distilled by adding 20 mL of sodium hydroxide (NaOH) into an Erlenmeyer flask with 15 mL 2% boric acid and titrated with 0.05N H_2_SO_4_.

### 4.4. Data Analysis

Analysis of variance was conducted to determine the effect of genotype, Zn application method, N level, and the interaction between the latter two factors using Statistix 9. All data were subjected to normality test by Shapiro–Wilk and data of grain Zn concentration were transformed by arcsine before being analysis. Least significant differences at *p* < 0.05 were used to compare mean values. The relationship between grain yield and grain N, Zn, and anthocyanin concentration was analyzed by using coefficient of correlation.

## 5. Conclusions

This study has established that grain yield and grain N, Zn, and anthocyanin concentration of the purple rice landraces responded differently to N and Zn application. The genotypes KAK and KS increased grain yield and anthocyanin simultaneously when N fertilizer was applied at a higher rate, but this effect was not observed for grain Zn. Foliar Zn application has been suggested as an effective tool to increase grain Zn concentration in all rice genotypes, but with different extent of increase among the genotypes. While soil Zn application should be carefully applied, as it could increase yield in some rice genotypes, it may reduce grain anthocyanin concentration. Therefore, improving grain anthocyanin and Zn concentrations among the purple rice genotypes could be managed through N fertilizer application, a common practice in rice crops. Zinc fertilizer application can improve grain anthocyanin and Zn concentration by proper selection of the N rate and Zn application method for the specific rice genotype. High grain N, Zn, and anthocyanin concentration in rice would help to promote human health by decreasing the risk of several serious diseases.

## Figures and Tables

**Table 1:** *p* values from analysis of variance (ANOVA) of grain yield, yield components and grain N, Zn, and anthocyanin among six purple rice genotypes grown under two N rates and three Zn application methods.

Characters	G	N	Zn	G × N	G × Zn	N × Zn	G × N × Zn
Yield and yield components							
Grain yield	<0.001	<0.001	<0.001	<0.05	<0.01	<0.001	ns
Tiller per plant	<0.001	ns	<0.001	ns	ns	ns	ns
Panicle per plant	<0.001	ns	<0.001	ns	<0.001	<0.001	ns
Spikelet number	<0.001	<0.001	<0.01	<0.05	<0.01	<0.001	ns
Number of filled grain	<0.001	<0.001	ns	ns	<0.01	<0.001	ns
100 grain weight	<0.001	ns	ns	ns	ns	ns	ns
Grain N, Zn, and anthocyanin							
Anthocyanin concentration	<0.001	<0.001	<0.001	<0.001	ns	<0.01	ns
Zn concentration	<0.001	ns	<0.001	<0.05	<0.001	ns	ns
N concentration	<0.001	<0.001	<0.001	<0.001	<0.05	ns	ns

ns represented non-significant difference at *p* < 0.05.

**Table 2:** Correlations between grain yield and grain N, Zn, and anthocyanin concentration of six purple rice genotypes.

Genotype	Anthocyanin	Zn	N
PIZ	−0.58 **	−0.05 ^ns^	−0.18 ^ns^
KAK	−0.06 ^ns^	−0.14 ^ns^	−0.52 *
KS	0.15 ^ns^	−0.21 ^ns^	0.12 ^ns^
KH-CMU	−0.21 ^ns^	−0.47 *	−0.48 *
KDK	0.23 ^ns^	0.06 ^ns^	0.35 ^ns^
HN	−0.62 **	−0.42 *	−0.12 ^ns^

*, and ** indicate significant correlations at *p* < 0.05, and 0.01, respectively, while ns represents non-significant difference at *p* < 0.05 (n = 6).

## Data Availability

No new data were created or analyzed in this study. Data sharing is not applicable to this article.
